# Editorial: New insights on bringing social context into addiction neuroscience

**DOI:** 10.3389/fnbeh.2024.1383016

**Published:** 2024-03-07

**Authors:** Olesya T. Shevchouk, Gayathri J. Dowling, Nicole L. Schramm-Sapyta

**Affiliations:** ^1^Institute of Neuroscience and Physiology, Department of Pharmacology, The Sahlgrenska Academy at the University of Gothenburg, Gothenburg, Sweden; ^2^National Institute on Drug Abuse (NIH), Bethesda, MD, United States; ^3^Duke Institute for Brain Sciences, Duke University, Durham, NC, United States

**Keywords:** race, ethnicity, family, prenatal, technology, social support, addiction, substance use

Addiction is a complex disease that has roots in genetics, brain function, the social environment, and their interactions. Studies to understand the mechanisms of substance use and addiction cannot occur in isolation. They need to be contextualized by the social environment and how it influences vulnerability to, or resilience against, substance use and substance use disorders. Thus, we are excited to present this Research Topic of Frontiers in Behavioral Neuroscience that deeply examines the Social Context of Addiction.

Although addiction is an illness as old as human society, our social context is constantly changing, and addiction with it. Social influences on substance use trajectories occur at various levels from society to neighborhood to family to the individual. Furthermore, the ubiquity of digital media exposure creates novel social contexts that may influence substance use and may be addictive themselves. Understanding the role of these social influences is critical as we strive to identify opportunities for clinical intervention.

Social factors and their effects are pervasive and long-lasting, and therefore complicated to measure and define. Longitudinal studies are needed, so we are glad to share many analyses from the Adolescent Brain Cognitive Development (ABCD) study, the most comprehensive longitudinal study of brain development and child health in the U.S. to date. The NIH-supported ABCD Study enrolled nearly 12,000 youth when they were 9–10 years old in 2016-2018 and is assessing them annually for at least 10 years, combining mental health, substance use, cultural, environmental, cognitive, and brain-scan information throughout adolescence. While participants were largely substance naïve at enrollment, substance use has been steadily increasing in the cohort as they mature (Lisdahl et al., 2021; Sullivan et al., 2022).

As you will see, the articles we have assembled represent a wide range of interpretations of “social context.” For example, Gebru et al., Peng et al., and Kohn et al. examine the role of the ever-present, always-complicated influences: family and parenting. Articles by Correa et al. and Bristol et al. specifically examine the roles that race and ethnicity play in vulnerability to substance use, examining a broad range of substances and alcohol, respectively. Articles by Shaheen et al. and Lu et al. examine technology addiction in cultural context with Arab and Chinese participants, respectively. Articles by Shaheen et al., Lu et al., and Peng et al. examine the addictive nature of technology itself (online gaming, internet use, and mobile phone use, respectively) and factors that increase risk or resilience for these forms of addiction.

These authors deftly tackle complicated mediating and moderating influences, showing, for example, that the effects of behavioral inhibition (Correa et al.) and family conflict (Bristol et al.) on predictors of substance use are moderated by racial and ethnic identity. The article by Peng et al. describes how individual responses to the environment influence drug taking by examining the effect of a future-time perspective and self-control.

Not surprisingly, many articles show the value of positive social support, whether it comes from peers or parents, in protecting against drug use (Lu et al.; Peng et al.; Bristol et al.; Kohn et al.). The pandemic, as we know, limited access to such supports, with detrimental effects, as shown by Shaheen et al.

The articles using data from the ABCD study provide a “teaser” to make us look ahead to future findings (Correa et al.; Bristol et al.; Gebru et al.). The analyses reported here examine the data through age 12, when most children have not yet initiated drug use. The authors are able to predict risk of future substance use based on decades of work identifying factors that are known predictors of drug use at this early age. Ongoing data collection is allowing researchers to follow these trajectories over time.

Much of the information presented in this Research Topic is actionable. Although we cannot change the fact that many parents suffer from mental health disorders, we can take steps to reduce conflict in the family environment (Gebru et al.; Bristol et al.). Even though some children will be exposed to cocaine or heroin prenatally, Kohn et al. show that the postnatal caregiving environment can greatly moderate their effects. Early recognition of behavioral inhibition can allow time for intervention to prevent anxiety, and its attendant risk of addiction (Correa et al.). Prevention efforts must be culturally sensitive to their racial/ethnic audiences (Correa et al.; Bristol et al.).

Taken together, this Research Topic provides an exciting update on the state of research in the social context of addiction. It provides actionable information and hope for prevention and treatment efforts.

## Author contributions

NS-S: Conceptualization, Writing—original draft, Writing—review & editing. OS: Conceptualization, Writing—review & editing. GD: Conceptualization, Writing—review & editing.
